# Editorial: Biomedical image segmentation and analysis

**DOI:** 10.3389/fphys.2023.1139288

**Published:** 2023-02-01

**Authors:** Naveen Aggarwal

**Affiliations:** UIET, Panjab University, Chandigarh, India

**Keywords:** biomedical imaging, noise removal, cancer classification, deep neural architecture, breast cancer, melanoma skin cancer, micro magnetic simulation, dysplasia

Advancements in the biomedical image capturing devices lead to improved multidimensional analysis with different perspectives. The availability of large amounts of data also made it possible to train deep learning models with a large number of parameters. These models provide better accuracy and help in automated segmentation and Region of Interest labeling for different medical applications. Researchers have proposed different deep learning architectures trained on large collection of images with a high accuracy and computational efficiency.

The efficacy of any Biomedical image segmentation technique depends upon the quality of images. The presence of noise such as speckle noise in Ultrasound images have a significant impact on segmentation. Its effect on final analysis has been highlighted in multiple studies. Li et al. proposed a novel 3D convolutional cloud based neural architecture to suppress the speckle noise in ultrasound images. The proposed approach is based on a guided filter algorithm that uses images preprocessed using spatial high pass filters. The suppression of noise helps in reduction of false recognition rate with good boundary wall retention for effective segmentation. But there is a need to analyze effects of such processing on structural and texture features.

To preserve the texture and frequency features, Moinuddin et al. proposed a network to upscale the resolution using different dilated convolutions and residual learning with different receptive fields. Further, to enhance the image quality while suppressing the speckle noise a novel encoder-decoder based UNET model with densely connected skip pathways is proposed. For better training of the network an augmented data set is used. The authors have proved that the proposed model not only removes the speckle noise but also reduces other noises and improves the overall quality of the images. However, efficacy of these models need to be tested on other modalities.

Deep Learning techniques are further explored by Gupta et al. on Gene Expression data to classify the different types of Cancers: Kidney renal clear cell carcinoma (KIRC), Breast Invasive Carcinoma (BRCA), lung adenocarcinoma (LUAD), Prostate Adenocarcinoma (PRAD) and Colon Adenocarcinoma (COAD). In their work, the RNA sequencing approach is used for assessing gene expression for effective bio molecular cancer diagnosis. The use of deep learning technique on microarray gene expression profiles is a unique way of classifying the tumors. The outcomes of the model can effectively be used for better treatment.

Ragab et al. used Deep Neural networks by taking inputs from a dermoscope to classify melanoma skin cancer. Dermoscope is used for taking images of skin lesions which are further segmented using melanocytic and nevus lesions. The resolution of input image is enhanced by removing certain artefacts pixels that make up hair and vessels. For segmentation, a level set approach is used without re-initialization by including reactive diffusion energy. For enhancing the lesions part, multiple morphological operations are used to make it distinct from surrounding skin. Final segmented part is enlarged and sigmoidal function is used to highlight the variances in the appearance of skin lesions.

The number of patients of breast cancer are increasing and about 20% of patients overexpress the HER2 gene. Lu et al. used 54 duplicated genes of trastuzumab and breast cancer from 521 target genes of the drug and 1,464 target genes of breast cancer. Finally, they got core genes such as GAPDH, MMP9, CCNA2, RRM2, CHEK1, etc. Using PPI network. Gene Ontology analysis clearly shows that the trastuzumab shows positive regulation of phospholipase activity, linoleic acid metabolic process, and negative regulation of endothelial cell proliferation. KEGG pathway analysis exhibited four pathways related to the formation and cure of breast cancer, containing Drug metabolism, Glutathione metabolism, Pyrimidine metabolism and PPAR signaling pathway. The binding abilities of the five core target proteins (GAPDH, MMP9, CCNA2, RRM2, CHEK1) is shown using molecular docking.

AI is playing a significant role during medical treatments. Motion tracking algorithms are now used with medical images for better contextual information in real time during treatments. Context specific features extracted using machine learning approaches can be used in tracking algorithms to recognize the deformed tissues. Qin et al. have used a tracking algorithm for the moving barium metal. They propose to process the video to improve the resolution and contrast enhancement. By taking inter frame differences, position calibration of tissues and organs can be done effectively. Further the use of Kernel Correlation Filter (KCF) and Discriminative Scale Space Tracker (DSST) correlation filtering improve the overall tracking results.

In this Research Topic, the challenging problem of stimulation of the vagus nerve is also addressed. Jeong et al. proposed to use micro-magnetic stimulation as an alternative to induce a focal stimulation for afferent fibers. The study was conducted by implanting the Micro-coils into the cervical vagus nerve in rats. Micro-magnetic stimulation caused only slight reduction in the respiration rate, whereas electrical VNS caused reduction of blood pressure, heart rate and respiration rate. To determine the optimal coil orientation profile, different EM simulations are conducted by estimating the strength and spatial distribution of the electric field. Micro-magnetic VNS clearly has an edge and can be used as an alternative. However, more such studies on a larger dataset needs to be conducted.

Bronchopulmonary dysplasia (BPD) is a common chronic lung disease and is very common in infants. If it is not detected at an early stage, it may affect the quality of life of a person. It is very necessary to explore the pathogenesis of BPD. For in depth understanding, there is a need to understand the mechanism of lncRNA in the formation of BPD. Another article in this Research Topic Bao et al. has explored to clarify the expression pattern of uc.375 in bronchopulmonary dysplasia and explore the interaction between uc.375 and FoxA1. It is observed that uc.375 plays an important role in the initiation and progression of BPD and negatively regulates FoxA1 expression, affecting alveolar development. This novel idea can be further explored to provide a treatment of BPD in a new direction.

Researchers have applied the state-of-the–art research methodologies on very challenging problems. The outcomes of all the Research Topics are very encouraging and can pave the way for future research in similar areas. Researchers have faced the challenge of data scarcity to prove the outcomes but more collaborative work can help us to overcome this as well.

